# The effects of land use types on microplastics in river water: A case study on the mainstream of the Wei River, China

**DOI:** 10.1007/s10661-024-12430-7

**Published:** 2024-03-08

**Authors:** Le Zhang, Xi Li, Qi Li, Xiaoqiang Xia, Hang Zhang

**Affiliations:** 1 College of Urban and Environmental Sciences, Northwest University, Xi’an, 710127 China; 2 Shaanxi Key Laboratory of Earth Surface System and Environmental Carrying Capacity, Xi’an, 710127 China

**Keywords:** Microplastics, Environmental factors, Land-use types, Raman spectroscopy, Wei River

## Abstract

Microplastics are widely found in rivers and their sediments, which will cause harm to the water ecological environment. The Wei River is a first-class tributary of the Yellow River, the fifth largest river in the world, and has vulnerable ecological environment and most sediment in the world. However, understanding how anthropogenic activities and environmental factors affect the microplastics distribution in this river is not clear. Based on this, the spatiotemporal distribution of microplastics in the Wei River were investigated. The abundance of microplastics ranged from 1033 to 8333 items/m^3^ and from 120 to 840 items/kg in the water and in the sediment, respectively. Fibers and fragments were the main shapes of Wei River, microplastics less than 500 μm were the main sizes, and black and white/transparent were the main colors. In Wei River, the abundance of microplastics in urban areas was higher than that in agricultural areas and mountainous areas. Furthermore, the correlation analysis revealed that microplastic abundance in the water was related to anthropogenic activities (population density, per capita GDP and distance) and environmental factors (water temperature, NH_3_-N, ORP), while in the sediments was correlated with anthropogenic activities (per capita GDP) and environmental factors (water temperature and NH_3_-N). This study reveals new patterns in microplastic pollution in the Wei River, underscoring the need for targeted environmental strategies. Our findings provide novel insights into the characteristics and distribution of microplastics, significantly adding to the current understanding of riverine microplastic pollution.

**Supplementary Information:**

The online version contains supplementary material available at 10.1007/s10661-024-12430-7.

## Introduction

Microplastics, defined as plastics less than 5 mm, are derived from two primary sources (Thompson et al., 2004). Firstly, they are directly added as ingredients in cosmetics and cleaning products. Secondly, plastic products undergo degradation and fragmentation as a result of external factors such as weathering, ultraviolet radiation, and physical wear (Wang et al., 2022). Microplastics are ubiquitous and have been detected in various environments, including the soil of the Qinghai-Tibet Plateau, the Arctic, the Antarctica and the Southern Ocean deep-sea sediments (Bergmann et al., 2017; Cunningham et al., 2020; Feng et al., 2021). Furthermore, microplastics have been found in organisms such as fish, birds and even human blood, highlighting their extensive distribution and potential for bioaccumulation (Kuhlman, 2022; Weitzel et al., 2021; Wootton et al., 2021). The difficult-to-degrade and transportable of microplastics has a significant ecological impact. Consequently, the pollution and ecological impact of microplastics have garnered substantial attention from the scientific community. This study expands on previous research by employing a detailed methodological approach, including comprehensive Raman spectroscopy analysis, to investigate microplastic pollution in the Wei River. This approach not only enriches the existing body of knowledge but also highlights the unique aspects of microplastic distribution in this specific riverine ecosystem.

The Wei River, in China is a first-class tributary of the Yellow River (the world's fifth largest river), holds the distinction of having the highest sediment load with an annual mean sediment discharge of 0.458 billion tons (Li et al., 2011). However, the health of the Wei River is a matter of grave concern, as indicated by its physical habitat assessment (Wang et al., 2019). Considering the significant role of the Wei River as a potential source of microplastics in the Yellow River, it becomes crucial to study microplastic pollution in this context. Rivers serve as major reservoirs for microplastics originating from land and also considered to be an important means of transportation for microplastics into the ocean, where they become persistent and challenging to remove once introduced (Shi et al., 2022). Microplastics may sink into the river sediments under the action of gravity, while the resuspension of microplastics from river sediments would cause secondary pollution to aquatic ecosystems (Huang et al., 2020). Therefore, comprehensive investigations focused on microplastics in rivers and river sediments are necessary.

Microplastic pollution represents a complex environmental issue influenced by a range of human-made and environmental factors (Wang et al., 2021). Previous studies have demonstrated that environmental factors, such as temperature, biochemical oxygen demand (BOD), total suspended substance (TSS) and turbidity, had the greatest influence on microplastics abundance in the Brantas River (Buwono et al., 2021). Various pathways contribute to the transport of microplastics from land to rivers, including runoff, rainstorms, atmospheric deposition, and sewage discharge from wastewater treatment plants, all of which are susceptible to human activities (Hurley et al., 2018; Oveisy et al., 2022; Trainic et al., 2020). Moreover, anthropogenic factors such as land use, urbanization have been reported to correlate strongly with microplastics abundance (Dai et al., 2022). The rapid economic development has led to the proliferation of cities and villages near rivers, resulting in changing land-use patterns due to human occupation, which in turn influences the input of terrestrial microplastics into rivers (Wang et al., 2019). Therefore, investigating the distribution of microplastics in river and their influence factors should be valuable for revealing the fates of microplastics.

Although the influence of environmental and anthropogenic factors on microplastics has been extensively studied, the factors driving the distribution of microplastics in different land use types remain poorly understood. In this study, sampling was carried out in the Wei River basin, which covers an area of 134,766 square kilometers, starting from its source and extending to the confluence with the Yellow River. The primary objective of this study were (1) to delineate spatial variations and composition of microplastics in both surface water and sediments, (2) to identify the effects of human activities (land use, population density, per capita GDP, distance from the central city) on the distribution of microplastics in the water and sediment, (3) to investigate and environmental factors (elevation, water temperature, DO, pH, conductivity, ORP, NH_3_-N, NO_3_^−^, TN, TOC) on the distribution of microplastics in the water and sediment. The findings from this study will shed light on the relationships between microplastics and human activities as well as environmental factors in the Wei River, providing valuable insights for the development of effective prevention and control measures against microplastic pollution. By unraveling the driving forces behind microplastic distribution in different land use types, this research will contribute to a more comprehensive understanding of microplastic pollution dynamics in river ecosystems.

## Materials and methods

### Study area and sample collection

**Fig. 1:**
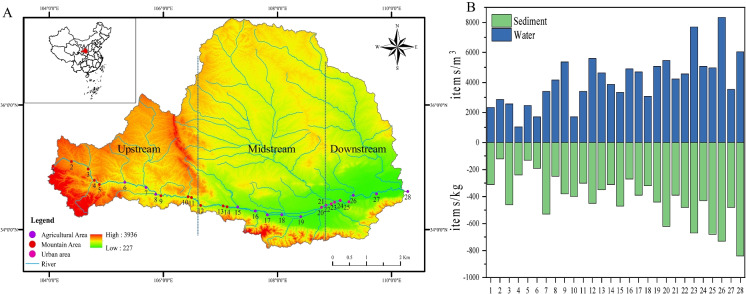
Sampling sites (A) location of Wei River and (B) the abundance of microplastics in the water and sediment

Wei River originates from Wei Yuan County, Ding Xi City and flows into the Yellow River at Tong Guan County, Wei Nan City. Wei River passes through the Gansu province and Shaanxi province, encompassing areas with high-density residential area. The upper sections mainly consist of mountainous areas, while the middle are predominantly agricultural areas. The lower sections are agricultural areas and urban areas. During the sampling period from September 23 to October 1, 2022, water and sediment samples were collected from 28 stations along Wei River (Fig. 1A). Detailed information about the geographical location of sampling sites is given in Table S1.

Thirty liters of water (0—30 cm in depth) was collected by a 5 L stainless steel tank at each site and then filtered in situ through a 20 μm stainless steel sieve. Residues retained on the sieve were rinsed into a glass bottle (250 mL) using pure water to form a concentrated water sample. Samples were transported to laboratory and stored at 4℃ until processed and analyzed. The sampling of sediment was collected about 2,000 g at 10—30 cm by stainless steel shovel and then packed in tin paper bag. A total of 28 water samples and 28 sediment samples were collected in this study.

### Extraction of microplastics

For the water sample, 30% H_2_O_2_ was added and shaken at 120 rpm at 60°C for 24 h to degrade the organic matter. Once the digestion was completed, the solutions were directly filtered onto a 0.45 μm (GF/F, 47 mmØ, Whatman) filter paper. Subsequently, the filter paper was placed in a petri dish and left to air dry naturally. Further examination was conducted using a stereo microscope. The sediment samples were naturally dried at room temperature (25°C). The sediment was then passed through a 2 mm sieve and microplastics larger than 2 mm are counted separately. A 100 g sample was weighed and placed in a clean 1,000 mL beaker for microplastic extraction. In this study, CaCl_2_ (1.5 g / cm^3^) and NaCl (1.2 g / cm^3^) solutions were used as flotation solutions.

A 250 ml of NaCl solution was poured into a glass beaker containing the sediment sample and stirred with a glass rod for at least 30 min. The mixture was then left undisturbed for 24 h, the supernatant was poured out, and the flotation process was repeated using 250 ml of CaCl_2_ solution to extract the microplastics from the sediment as completely as possible. Finally, the supernatant was carefully poured out and the process was repeated for the water samples.

### Inspection and identification of microplastics

A stereo microscope (SMZ25, Nikon, Japan) was used to inspect the particles on the air-dried filter membrane, and counted them according to shape (fiber, film, fragment, granule), size (< 50 μm, 50—100 μm, 100—500 μm, 0.5—1 mm, 1—2 mm, 2—5 mm), and color (black, white/transparent, blue, red, yellow, green). The total number of microplastics at each sampling site, as well as the number of microplastics of each color, size, shape was recorded.

To identify certain particles, Raman spectroscopy was used. Suspect particles extracted from the sample were placed on a glass slide using an incident laser beam of 532 nm with a scan time of 10 s and a laser power of 100 mW, while the scattered light intensity at wavelengths of 100 to 3200 cm^−1^ was recorded. The spectra obtained are then compared with standard spectra and the polymer type is determined based on the location of characteristic peaks.

### Quality control measures

During the process of microplastic collection, steel sieves, iron shovel, glass sampling bottles were thoroughly cleaned with pure water prior to use. For microplastic analysis, all instruments utilized in the experiments were cleaned with pure water, and researchers wore latex gloves and cotton lab coats. To ensure the integrity of the samples, they were covered with aluminum foil during the static process. To verify the absence of contamination, a 24—hour filter membrane placed in the laboratory yielded no detected microplastics, and no plastic particles were examined after passing 30 L of distilled water through the filter paper under vacuum filtration.

### Acquisition of other data

Per capita gross domestic product (GDP) and population density data were summarized on the websites of the county (district) government and are displayed in Table S2. The data of water temperature, dissolved oxygen (DO), potential of hydrogen (pH), conductivity, redox potential (ORP) was measured by portable water quality parameter detector (LH—T600) on site and are displayed in Table S3. The data of NH_3_-N (HJ/T 195—2005), NO_3_^−^ (HJ/T 197—2005), total nitrogen (TN) (HJ/T 199—2005), total organic carbon (TOC) (HJ 501—2009) was measured in the laboratory and are displayed in Table S3. The abundance of microplastics in water (items/m^3^) is obtained by dividing the number of detected microplastics by the volume of water (Lin et al., 2021). The abundance of microplastic in the sediment (items/kg) was involved dividing the number of detected microplastics by the weight of sediment.

### Statistical analysis

The spatial distribution of sampling points was geographically mapped using ArcGIS10.5 according to the longitude and latitude collected on site. One-way analysis of variance (ANOVA) was performed to analyze the differences in microplastic abundance among different land use types and geographical locations (p < 0.05). Principal component analysis (PCA) was used Origin (Version 2021) software to investigate the underlying factors contributing to the significant differences in microplastics among different land use and geographical locations. Additionally, a comparison of microplastic sizes between the water and sediment using ANOVA (p < 0.05). Spearman correlation analysis was employed in IBM SPSS software (Version 21) to examine the correlations between variables, and the results were visualized using Origin software (Version 2021).

## Results and discussion

### Abundance and distribution of microplastic

In this work, microplastics were observed in all water and sediment samples in this study, indicating the prevalence of microplastics in the Wei River. The concentration of particles of water varied from 1033 to 8333 items/m^3^ with a mean of 4145 ± 1700 items/m^3^. The minimum and maximum abundance of microplastics in the water were detected at site 4 (1033 items/m^3^) and site 26 (8333 items/m^3^), respectively (Fig. 1B). In the sediment, the abundance of microplastics ranged from 120 to 840 items/kg with an average of 415 ± 176 items/kg. Different from the distribution of microplastics in water, the minimum and maximum values of microplastics in the sediment were found at site 2 (120 items/kg) and site 28 (840 items/kg), respectively (Fig. 1B). The sampling sites encompassed mountainous, agricultural and urban areas and the intensity of anthropogenic activities varied among the sampling sites, resulting in significant variation in their distribution patterns with abundances. Sites 2 and 4 were located in the mountainous areas of the upper Wei River, where the population density and per capita GDP were low. On the contrary, since microplastics can be transferred horizontally downstream (Yan et al., 2021), site 26, located in the agricultural area, which was the first site downstream of the urban area, had the highest abundance of microplastics in water. However, the sampling site with the highest microplastic abundance in sediment samples was site 28 located in the agricultural area, probably because site 28 is located at the confluence of the Wei River and the Yellow River, where the riverbed widens and the flow velocity slows down, making it easier for microplastics to settle, and at the same time, due to the transportation of sediment, resulting in the ease of microplastics to be wrapped up in the sedimentation. It can be seen that the abundance of microplastics in the Wei River has obvious spatial specificity, and this uneven distribution may be caused by anthropogenic activities and environmental factors (Wang et al., 2021).

Previous studies by Ding et al. (2019) have shown that the abundance of microplastics was 3670—10,700 items/m^3^ in the water and 360—1320 items/kg in the sediment of the Wei River. Zhang et al. (2023) found that the abundance of microplastics in the water and sediments of the Wei River ranged from 2960 to 10,320 items/m^3^ and 4.774 × 10^5^—6.163 × 10^6^ items/m^3^, respectively. The microplastic abundance in the present study were lowest compared to the study of Ding et al. (2019) and Zhang et al. (2023). We speculate that this may be due to the fact that the present study sampled during the wet season (September), and the increased water volume and better hydraulic conditions than other periods made it easier for microplastics to be carried downstream, resulting in lower microplastic abundance (Nel et al., 2018). Conversely, microplastics are more easily retained and settled during dry water periods. Therefore, we suggest that microplastics in the Wei River during different periods be studied afterwards. *Microplastic characterization.*

**Fig. 2:**
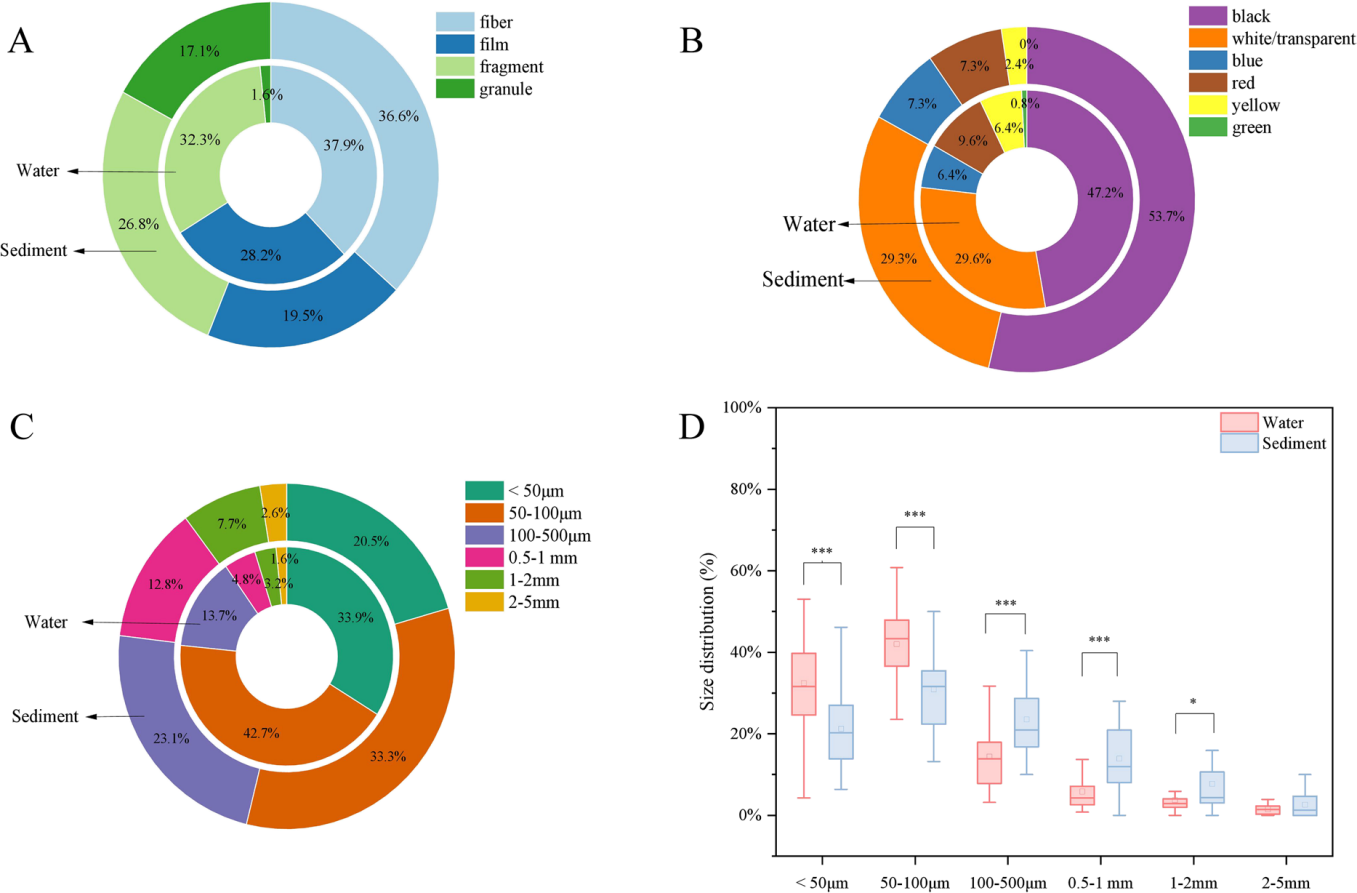
The (A)shape, (B) color, and (C) size distribution of microplastics in the water and sediment. (D) Comparison of differences in distribution between the water and sediment. * p < 0.05, ** p < 0.01, *** p < 0.001

The shape, color and size characteristics and chemical composition of microplastics can provide clues to their potential sources. Microplastics were classified into four shapes (fiber, film, fragment, granule), six color classifications (black, white/transparent, blue, red, yellow, green), and six size categories (< 50 μm, 50—100 μm, 100—500 μm, 0.5—1 mm, 1—2 mm, 2—5 mm) to provide a comprehensive understanding of the microplastics in this study area. The representative composition of microplastics in terms of shape, color, and size was illustrated in Figs. 2A, 2B, and 2C, respectively.

Among the different shapes of microplastics observed in the water, fibers were the most common, accounting for 37.9% of the total, followed by fragments (32.3%), films (28.2%), and granules (1.6%). Similarly, fibers (36.6%) and fragments (26.8%) were the most common in the sediments, followed by films (19.5%), while granular plastics accounted for 17.1% of the microplastics in the sediments (Fig. 2A). Fibrous microplastics were found to be the predominant form of microplastics in this study, and similar findings were found by Lin et al. (2018) in the Pearl River. Fibers are widely used in daily life to make various materials such as textiles, and their abrasion during washing releases a large number of microplastics into wastewater, and some studies have shown an average emission rate of 18,000,000 synthetic microfibers with a reference load of 6 kg synthetic fibers (Gago et al., 2018; Galvao et al., 2020; Pinlova et al., 2022). In addition, films and fragments make up a significant proportion of the Wei River, generally originating from weathering and degradation of mulch used in agricultural production and plastic waste from daily life (Yang et al., 2021). However, the Wei River had the least amount of granular microplastics, which may be due to the smaller size and larger specific surface area of granular microplastics, which can easily adsorb sludge and thus be transferred to the sludge in the WWTP (Liu et al., 2019).

The microplastics found in the Wei River exhibited a diverse range of colors. Figure 2B shows that black (47.2%) and white/transparent (29.6%) were the most common colors in microplastics in the Wei River, followed by red (9.6%), blue (6.4%), and yellow (6.4%). Similarly, black (53.7%) and white/ transparent (29.3%) microplastics were the most common colors in sediments, followed by red (7.3%), blue (7.3%), and yellow (2.4%). However, green microplastics were not detected in the sediments. We hypothesized that the microplastics of different colors in the Wei River might originate from plastic products of different colors. In this study, the reason that black color accounted for the most may be because black plastic film is widely used to cover cultivated land due to its good thermal insulation properties (Zhao et al., 2023). White/transparent microplastics are widely used in production and daily life, such as white plastic bags, packages, bottles, and agricultural mulch films (Yang et al., 2021). In addition, fading of colored microplastics is another potential source of white/clear microplastics (Pan et al., 2020). Different organisms have different preferences for different colored microplastics, e.g., goldfish (Carassius auratus) prefers green and black microplastics and sea cucumbers prefer black plastics (Mazlan et al., 2023; Xiong et al., 2019). Colored microplastics are more likely to be ingested by aquatic organisms, so there is a need to investigate the color of microplastics (Rios et al., 2022).

In our study, small-sized microplastics (< 500 μm) dominated both in the water and sediments, accounting for 90.4% and 76.9% of the total, respectively (Fig. 2C). Small-sized microplastics may be formed by the combined effects of hydraulic shear and weathering of plastics, or they may come directly from cosmetic additives(Li et al., 2022; Zhou et al., 2023). Due to their large specific surface area, small-sized microplastics absorb more toxic and hazardous substances, thus causing greater harm to the environment (Liu et al., 2020). In addition, to compare the size differences of microplastics between the water and sediment compartments, an analysis of variance (ANOVA) was conducted. This study found that the proportion of microplastics (< 100 μm) in water was significantly higher than that in sediments (Fig. 2D), unlike Xia et al. (2022) who found that smaller (50–100 μm) and larger (500—5000 μm) microplastics were more likely to accumulate in sediments. We hypothesize that the reason for this result may be due to the fact that microplastics (< 100 μm) take longer to settle and that the settled microplastics will be resuspended in the water due to hydrodynamic perturbations (Eo et al., 2019).

**Fig. 3:**
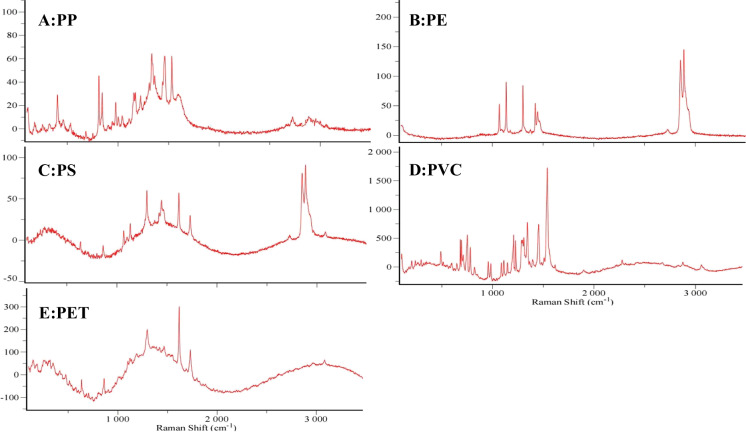
Selected Raman spectra. A: Polypropylene (PP); B: polyethylene (PE); C: polystyrene (PS); D: polyvinyl chloride (PVC); E: polybutylene terephthalate (PET)

**Fig. 4:**
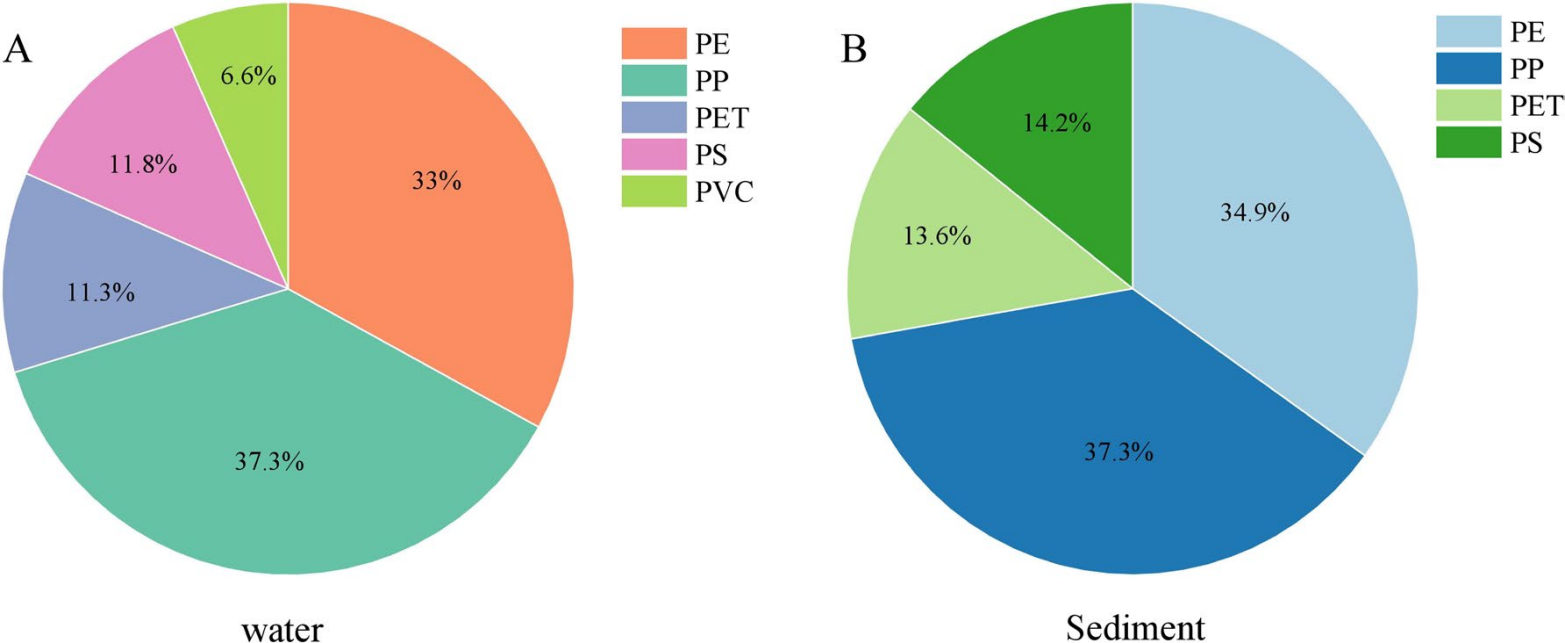
Polymer compositions of microplastics found (A) in the water and (B) in the sediment

Raman spectroscopy was used to identify the types of microplastics. In this study, a total of 417 randomly selected particles from water samples and 305 randomly selected particles from sediments were analyzed. 212 particles from water samples and 182 particles from sediments were identified as microplastics. Five polymer types of microplastics were detected including polypropylene (PP), polyethylene (PE), polystyrene (PS), polybutylene terephthalate (PET) and polyvinyl chloride (PVC). The Raman spectra are shown in Fig. 3. The percentages of the five microplastics (PP, PE, PS, PET and PVC) in the water samples were 37.3%, 33%, 11.8%, 11.3% and 6.6%, respectively (Fig. 4A). In contrast, only four polymers (PP, PE, PS, and PET) were detected in the sediment with 37.3%, 34.9%, 14.2%, and 13.6%, respectively, and no PVC was detected (Fig. 4B). In terms of the types of microplastics, PP and PE were the most common microplastics in the Wei River. PP and PE are widely used in textile and agricultural mulch films and are commonly found in municipal domestic waste and refuse, which may enter rivers through effluent discharges from wastewater treatment plants or surface runoff (Chu et al., 2023).

### Environmental and anthropogenic factors affecting the distribution of microplastics.

**Fig. 5:**
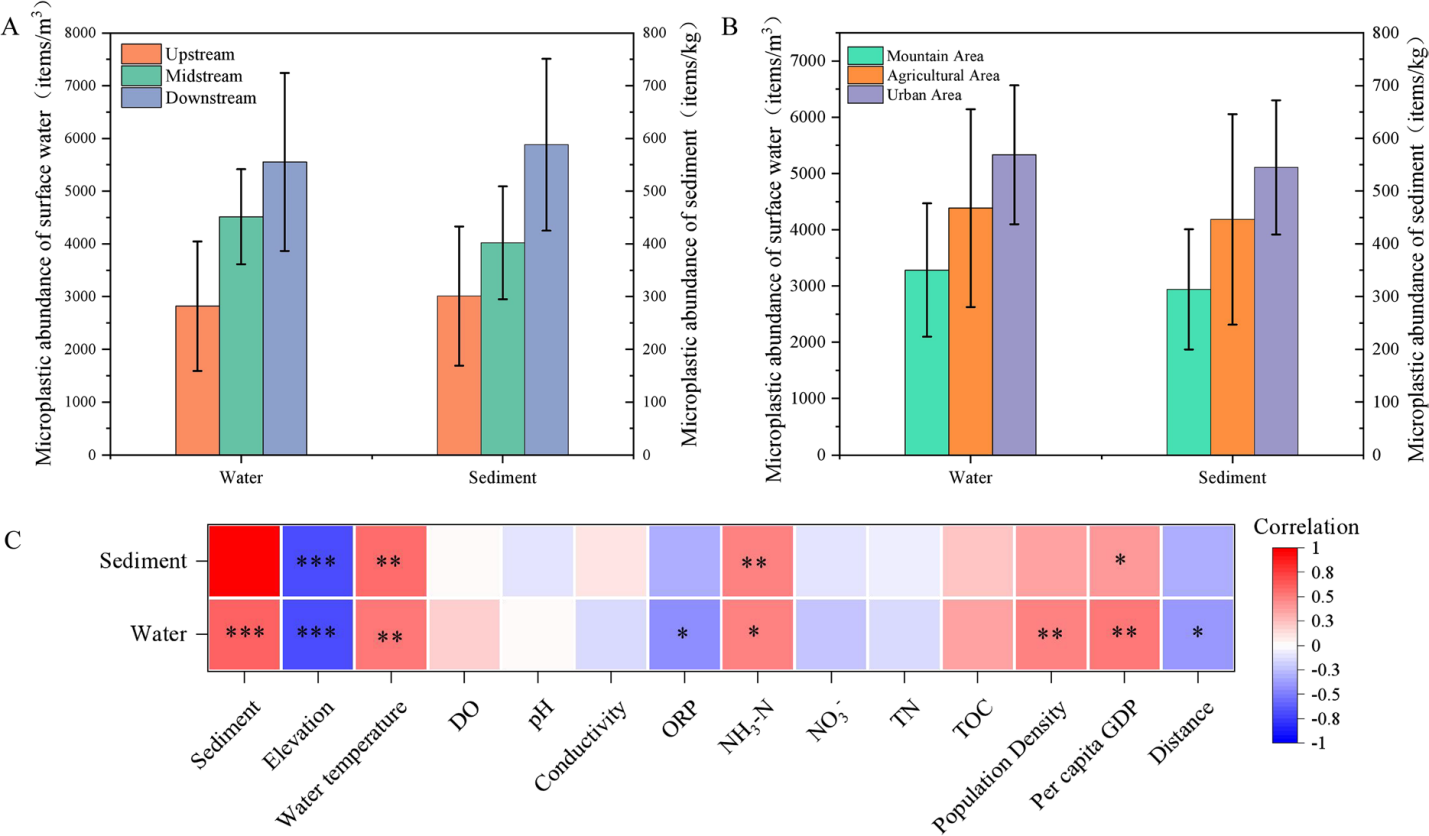
The relationship between microplastic abundance with (A) geographical location and (B) land use of the sampling sites. (C) Spearman’s correlations of microplastics abundance with human factors (elevation, population density, per capita GDP, distance from the central city) and environmental factor (water temperature, DO, pH, conductivity, ORP, NH_3_-N, NO_3_^−^, TN, TOC). * p < 0.05, ** p < 0.01, *** p < 0.001

In our study, microplastics in both the water and sediments exhibited a positive correlation with ammonia nitrogen (NH_3_-N) and water temperature, and a negative correlation with elevation (Fig. 5C). These findings align with previous research, including a study by Buwono et al., (2021), which demonstrated a direct relationship between microplastic abundance and water temperature in the Brantas River, East Java, Indonesia. Similarly, research conducted by Shekoohiyan and Akbarzadeh (2022) found a positive correlation between microplastic abundance and NH_3_-N in the Jajroud River of Tehran, and Zhou et al. (2021) who showed that microplastic abundance in the Yangtze River soils was negatively correlated with elevation. We hypothesize that the association between ammonia nitrogen (NH_3_-N) and microplastic contamination could be attributed to their potential origin from the same contamination sources, such as domestic wastewater and agricultural production. This suggests a potential link between the presence of microplastics and ammonia nitrogen contamination, a connection that has been highlighted in recent research such as the study conducted by Xu et al. (2023). Temperature plays a role in accelerating the fragmentation of rigid plastic products. In various contexts, such as the breakdown of mulch used in agricultural production or the degradation of plastic bags and packaging in everyday life, higher temperatures can hasten the process (Singh & Sharma, 2008). This may explain the observed association between water temperature and microplastic pollution in both water and sediments in our study, as increased temperatures could lead to a quicker breakdown of these materials into microplastics. Additionally, we found a significant correlation between the abundance of microplastics in both water and sediment, and the elevation of the region. We hypothesize that this may be due to the gradual transition of the study area from upstream to midstream and downstream, from mountainous areas to agricultural and urban areas, with a gradual increase in population density. In addition, another of our hypotheses is that this may result from the migration of similar types of microplastics from upstream to downstream areas, as noted in research by Yan et al. (2021). Considering that the Wei River is a tributary of the Yellow River, a comprehensive survey of microplastics within the Wei River basin serves as a foundational basis for a broader investigation into microplastic contamination within the entire Yellow River Basin.

For anthropogenic factors, our study found positive correlations between the abundance of microplastics in the water and both population density and per capita GDP, while a negative correlation was observed with the distance from the central city (Fig. 5C). This pattern aligns with previous research, which observed connections between anthropogenic factors within catchment areas and microplastic pollution (Chen et al., 2020). Our hypothesis is that areas with a dense concentration of human activities and higher economic levels tend to produce a significant amount of microplastic pollution. In contrast, in sediment samples, the abundance of microplastics was found to be significantly and positively correlated only with per capita GDP (Fig. 5C). This discrepancy might be explained by the fact that microplastics in sediments are influenced not just by human factors but also by elements affecting the deposition of microplastics in water. This includes variables such as the density differences between freshwater and microplastic particles, which can affect how microplastics settle and accumulate.

**Fig. 6:**
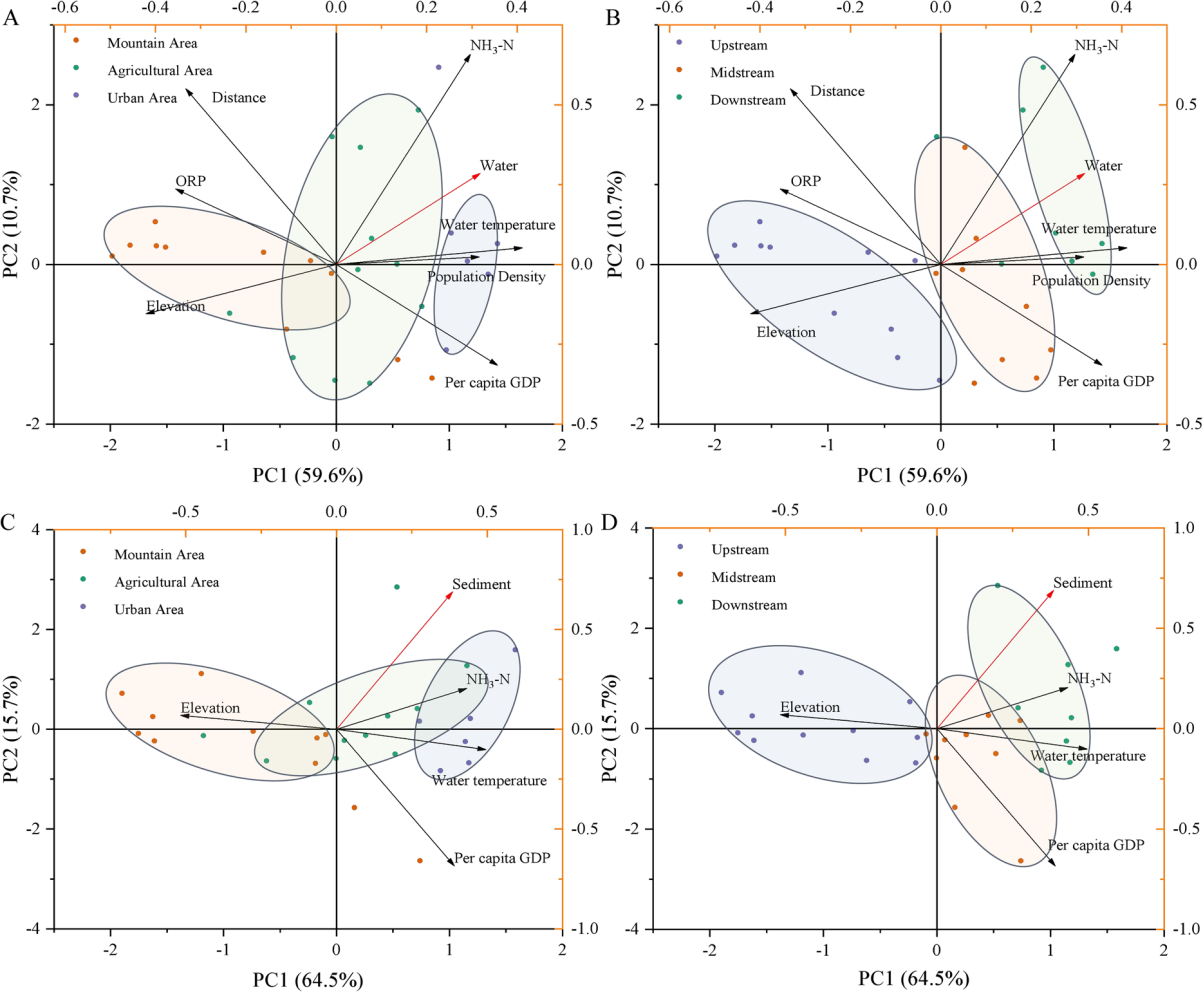
Principal component analysis based on different land use (A. in the water and C. in the sediment) and geographical locations (B. in the water and D. in the sediment)

Human activities, which lead to changes in land use, vary in intensity across the upper, middle, and lower reaches of the Wei River, as described by Wang et al. (2019). In our study, we categorized the sampling sites geographically into upstream, midstream, and downstream locations, and by land use into mountainous, agricultural, and urban areas. This allowed us to examine the distribution of microplastics across different landscapes and geographic location (Fig. 5A and 5B). Specifically, the average abundance in urban areas was measured at 5333 items per cubic meter (items/m^3^) in water and 545 items per kilogram (items/kg) in sediment. In mountainous areas, the corresponding figures were 3258 items/m^3^ in water and 313 items/kg in sediment. For agricultural areas, the average abundance was found to be 4385 items/m^3^ in water and 446 items/kg in sediment. A PCA was conducted to analyze the environmental and anthropogenic factors for the different land use types (urban areas, agricultural area and mountain areas) in the study area, and the total interpretations of principal component 1 and principal component 2 were 70.3% and 80.2% for the water and sediments, respectively (Fig. 6A and 6C). Frequent human activities in urban areas have led to an increase in microplastic content in domestic wastewater and the indiscriminate disposal of plastic waste. Compared to the average microplastic abundance under different land uses, the microplastic abundance in the agricultural area was intermediate and showed a higher pattern than in the mountainous area. Presumably because agricultural areas generally have lower population densities, less rapid economic development and lower consumption of plastics products compared to urban areas. In addition, agricultural land use is more likely to retain microplastic particles than urban land use, due to permeable surface and lower rates of overland flow (Horton & Dixon, 2017).

## Conclusion

In conclusion, this study provides distinctive insights into the microplastic pollution of the Wei River, differentiating it from existing research in significant ways. Our findings highlight the heterogeneity of microplastic distribution and emphasize the need for region-specific strategies in managing this global environmental issue. The study uncovers the spatial distribution of microplastics in a typical sediment-laden river system, specifically examining differences in microplastic abundance across various segments of the Wei River Basin, including mountainous, agricultural, and urban areas, as well as upstream, midstream, and downstream locations. Among the land use types analyzed, urban areas exhibited the highest microplastic abundance in both water and sediment.

Our findings also indicate a trend of increasing microplastic concentrations from upstream to downstream, which suggests potential migration from the Wei River to the Yellow River, and ultimately into the ocean. This pattern appears to result from the combined influence of environmental and anthropogenic factors. Specifically, in water, microplastic abundance correlated with human activities such as population density and per capita GDP, as well as environmental factors like elevation, water temperature, NH_3_-N, and ORP. In sediments, correlations were observed with per capita GDP and environmental factors such as water temperature and NH_3_-N.

The majority of plastics enter the environment due to mismanagement, emphasizing the need for more effective recycling practices and control over anthropogenic discharges. The insights garnered from this study hold significant implications for managing and preventing microplastic pollution, not only within the Wei River Basin but also in other regions facing similar challenges.

## Data availability

Data will be made available on request.

### Supplementary Information

Below is the link to the electronic supplementary material.

Supplementary file1 (DOCX 40 KB)

## Declarations

All authors have read, understood, and have complied as applicable with the statement on "Ethical responsibilities of Authors" as found in the Instructions for Authors.
