# Supplementary material for: The effects of land use types on microplastics in river water: A case study on the mainstream of the Wei River, China
Source: Environ Monit Assess. 2024 Mar 8;196(4):349. doi: 10.1007/s10661-024-12430-7 (PMC10920460; doi:10.1007/s10661-024-12430-7)
Supplement: Supplementary file 1 — Supplementary file1 (DOCX 40 KB) [file 10661_2024_12430_MOESM1_ESM.docx]

**Supplementary Data**

The effects of land use types on microplastics in river water: A case study on the mainstream of the Wei River, China

Le Zhang ^a,b,1^, Xi Li ^a,b,1^, Qi Li ^a,b,*^, Xiaoqiang Xia ^a,b^, Hang Zhang ^a,b^

*a College of Urban and Environmental Sciences, Northwest University, Xi’an, 710127，China*

*b Shaanxi Key Laboratory of Earth Surface System and Environmental Carrying Capacity, Xi’an, 710127，China*

Submitted to *Environmental Monitoring and Assessment*

Table S1. Sampling site and microplastic abundance of the Wei River

Table S2. Anthropogenic factors of the sampling sites

Table S3. Water quality of the sampling sites

Table S1. Sampling site and microplastic abundance of the Wei River

| Sampling site | Water (items/m^3^) | Sediment  (items/kg) | Longitude  (°E) | Latitude (°N) | Elevation  (m) |
| --- | --- | --- | --- | --- | --- |
| 1 | 2333 | 310 | 104.066811 | 35.020232 | 2397.945933 |
| 2 | 2867 | 120 | 104.390453 | 35.092579 | 1917.339676 |
| 3 | 2567 | 460 | 104.682676 | 34.972657 | 1693.421350 |
| 4 | 1033 | 240 | 104.790666 | 34.791703 | 1541.022448 |
| 5 | 2467 | 130 | 104.879910 | 34.725010 | 1468.067148 |
| 6 | 1700 | 190 | 105.325343 | 34.760752 | 1276.971809 |
| 7 | 3400 | 530 | 105.696489 | 34.675031 | 1136.971065 |
| 8 | 4167 | 250 | 105.866687 | 34.566655 | 1089.323308 |
| 9 | 5367 | 380 | 105.957919 | 34.540763 | 1070.354348 |
| 10 | 1700 | 400 | 106.434928 | 34.525923 | 901.467939 |
| 11 | 3400 | 300 | 106.495332 | 34.513026 | 899.115446 |
| 12 | 5600 | 450 | 106.654892 | 34.386770 | 792.231836 |
| 13 | 4633 | 350 | 107.049885 | 34.378973 | 604.039233 |
| 14 | 3867 | 310 | 107.118330 | 34.365059 | 591.552016 |
| 15 | 3333 | 470 | 107.304831 | 34.362951 | 550.640528 |
| 16 | 4900 | 270 | 107.613121 | 34.298012 | 501.724650 |
| 17 | 4700 | 390 | 107.824320 | 34.234769 | 468.841283 |
| 18 | 3067 | 320 | 108.077026 | 34.236347 | 436.045904 |
| 19 | 5067 | 440 | 108.409834 | 34.206323 | 401.796942 |
| 20 | 5467 | 620 | 108.774016 | 34.356706 | 378.788930 |
| 21 | 4233 | 390 | 108.850400 | 34.390367 | 373.967528 |
| 22 | 4567 | 480 | 108.944863 | 34.411904 | 365.392634 |
| 23 | 7700 | 670 | 109.004214 | 34.441021 | 359.131786 |
| 24 | 5067 | 430 | 109.102002 | 34.466751 | 358.485523 |
| 25 | 4967 | 680 | 109.254245 | 34.446057 | 348.785135 |
| 26 | 8333 | 730 | 109.332180 | 34.549010 | 348.926292 |
| 27 | 3533 | 480 | 109.737731 | 34.571741 | 331.683941 |
| 28 | 6033 | 840 | 110.282933 | 34.610980 | 324.095138 |

Table S2. Anthropogenic factors of the sampling sites

| Sampling site | Population Density  (people/km^2^) | Per capita GDP (billon Yuan) | Distance  (km) |
| --- | --- | --- | --- |
| 1 | 133.50 | 4.61 | 80.32 |
| 2 | 133.50 | 4.61 | 58.53 |
| 3 | 176.50 | 8.74 | 70.15 |
| 4 | 178.97 | 7.11 | 88.67 |
| 5 | 178.97 | 7.11 | 80.19 |
| 6 | 317.15 | 8.76 | 42.5 |
| 7 | 157.91 | 19.37 | 11.72 |
| 8 | 157.91 | 19.37 | 12.06 |
| 9 | 157.91 | 19.37 | 21.32 |
| 10 | 190.43 | 24.14 | 76.31 |
| 11 | 190.43 | 24.14 | 69.72 |
| 12 | 190.43 | 24.14 | 53.57 |
| 13 | 1492.97 | 40.03 | 17.57 |
| 14 | 638.86 | 58.78 | 10.47 |
| 15 | 190.43 | 24.14 | 7.2 |
| 16 | 418.29 | 17.47 | 36.03 |
| 17 | 325.50 | 19.98 | 55.14 |
| 18 | 1865.67 | 15.78 | 59.26 |
| 19 | 990.93 | 28.10 | 30.82 |
| 20 | 1112.50 | 30.65 | 8.08 |
| 21 | 1112.50 | 30.65 | 15.67 |
| 22 | 1112.50 | 30.65 | 25.68 |
| 23 | 1556.12 | 38.47 | 13.78 |
| 24 | 1556.12 | 38.47 | 21.63 |
| 25 | 744.59 | 26.01 | 32.9 |
| 26 | 744.59 | 26.01 | 42.08 |
| 27 | 329.62 | 19.25 | 26.44 |
| 28 | 235.17 | 4.74 | 77.58 |

Table S3. Water quality of the sampling sites

| Sampling site | Water temperature( ℃ ) | DO (mg/L) | pH | Conductivity(μS/cm） | ORP (mV) | NH_3_-N (mg/L) | NO_3_^-^  (mg/L) | TN  (mg/L) | TOC  (mg/L) |
| --- | --- | --- | --- | --- | --- | --- | --- | --- | --- |
| 1 | 15.2 | 8.11 | 8.39 | 336 | 233.1 | 0.058 | 2.11 | 2.47 | 4.008 |
| 2 | 15.4 | 8.08 | 8.42 | 469 | 237.5 | 0.142 | 3.61 | 4.11 | 4.728 |
| 3 | 15.2 | 8.14 | 8.38 | 1001 | 225.3 | 0.121 | 6.18 | 6.34 | 5.778 |
| 4 | 12.8 | 8.35 | 8.32 | 2230 | 218.6 | 0.092 | 14.85 | 15.96 | 7.409 |
| 5 | 13.8 | 8.77 | 8.41 | 1018 | 227.9 | 0.121 | 5.88 | 7.00 | 4.919 |
| 6 | 16.4 | 8.19 | 8.38 | 974 | 180.7 | 0.156 | 7.74 | 9.02 | 5.092 |
| 7 | 20.8 | 7.73 | 8.42 | 1145 | 200.3 | 0.124 | 8.14 | 9.29 | 6.395 |
| 8 | 19.4 | 7.90 | 8.45 | 1389 | 130.1 | 0.150 | 7.17 | 9.27 | 5.649 |
| 9 | 16.4 | 8.07 | 8.33 | 1151 | 191.5 | 0.098 | 6.53 | 8.37 | 7.28 |
| 10 | 19.1 | 7.98 | 8.34 | 991 | 177.8 | 0.228 | 4.84 | 7.99 | 5.247 |
| 11 | 20.8 | 7.87 | 8.47 | 897 | 145.3 | 0.217 | 4.56 | 6.57 | 8.205 |
| 12 | 20.1 | 7.95 | 8.51 | 851 | 154.6 | 0.147 | 5.07 | 6.53 | 8.766 |
| 13 | 20.4 | 12.12 | 8.70 | 682 | 188.7 | 0.136 | 4.66 | 5.90 | 5.396 |
| 14 | 20.7 | 10.54 | 8.48 | 703 | 159.9 | 0.338 | 4.88 | 6.29 | 6.52 |
| 15 | 22.8 | 9.04 | 8.38 | 694 | 145.6 | 0.156 | 4.55 | 6.56 | 6.618 |
| 16 | 23.3 | 8.45 | 8.19 | 598 | 168.2 | 0.182 | 0.85 | 3.48 | 53.243 |
| 17 | 20.9 | 8.78 | 8.19 | 697 | 166.3 | 0.488 | 7.18 | 9.17 | 27.492 |
| 18 | 22.9 | 11.78 | 8.57 | 644 | 163.7 | 0.208 | 6.43 | 8.49 | 9.172 |
| 19 | 24.1 | 10.44 | 8.54 | 685 | 138.5 | 0.199 | 7.14 | 8.87 | 7.051 |
| 20 | 23.3 | 10.41 | 8.66 | 659 | 134.7 | 0.197 | 6.63 | 7.83 | 13.163 |
| 21 | 28.4 | 16.04 | 8.73 | 628 | 119.9 | 0.451 | 5.46 | 6.60 | 7.769 |
| 22 | 26.7 | 10.03 | 8.29 | 780 | 162.5 | 0.434 | 6.55 | 8.22 | 5.198 |
| 23 | 22.5 | 9.36 | 8.54 | 678 | 148.1 | 0.416 | 4.69 | 7.98 | 5.577 |
| 24 | 22.6 | 10.05 | 8.23 | 685 | 150.4 | 0.431 | 4.12 | 7.13 | 5.831 |
| 25 | 22.5 | 9.16 | 8.33 | 1076 | 176.2 | 0.847 | 3.70 | 6.21 | 8.27 |
| 26 | 23.2 | 8.61 | 8.38 | 972 | 206.4 | 0.457 | 3.91 | 6.21 | 6.606 |
| 27 | 24.6 | 7.52 | 8.33 | 1586 | 138.2 | 0.364 | 5.45 | 7.06 | 8.455 |
| 28 | 21.4 | 7.63 | 8.20 | 1012 | 147.6 | 0.260 | 3.76 | 4.18 | 5.701 |
